# ROLE OF SOCIAL DETERMINANTS IN ENROLLMENT AND DISENROLLMENT IN MEDICARE INSURANCE PLANS IN OLDER MEXICAN AMERICANS

**DOI:** 10.1093/geroni/igz038.2081

**Published:** 2019-11-08

**Authors:** Amit Kumar, Maricruz Rivera-Hernandez, Lin-Na Chou, Amol Karmarkar, Yong-Fang Kuo, Kenneth J Ottenbacher

**Affiliations:** 1 Northern Arizona University, Flagstaff, Arizona, United States; 2 Brown University, Providence, Rhode Island, United States; 3 University of Texas Medical Branch, Galveston, TX, Galveston, Texas, United States

## Abstract

Objective: The objective of this study is to examine the association between social-medical risk factor with disenrollment from Medicare Fee-for-Service (FFS) and enrollment in a Medicare Advantage (MA) plan in Older Mexican Americans. Methods: The sample included older adults participating in the Hispanic Established Populations for the Epidemiologic Study of the Elderly linked with Medicare data. We used logistic regression to estimate odds ratios (OR) for the association of each sociodemographic and clinical factor with insurance plan switching. Results: FFS enrollees were more likely to speak Spanish, less educated, lower income, disability, and be dual eligible compared to MA enrollees. At 2-year follow up, older adults with social support had higher odds of switching from FFS to MA after controlling for all covariates (OR; 1.73, 95% CI: 1.11-2.69). Conclusion: Having social support from family and the community was strongly associated with disenrollment from FFS and transition to an MA plan.

